# A “tetra‐axial” system in transfemoral lead extraction

**DOI:** 10.1002/ccr3.983

**Published:** 2017-05-10

**Authors:** Tsuyoshi Isawa, Takashi Yamada, Taku Honda, Kazuhiro Yamaya, Tatsushi Ootomo

**Affiliations:** ^1^ Department of CardiologySendai Kousei HospitalSendaiJapan; ^2^ Department of CardiologyTakaishi Fujii Cardiovascular HospitalTakaishiJapan; ^3^ Department of Cardiovascular SurgerySendai Kousei HospitalSendaiJapan

**Keywords:** A tetra‐axial system, child‐in‐mother technique, lead extraction

## Abstract

Pacemaker lead extractors must become familiar with transfemoral approaches for lead extraction as a bail‐out procedure for a failed superior approach. We presented a “tetra‐axial” system for transfemoral lead extraction. This system would be more widely applicable in cases with difficulties in extraction, resulting in more procedural success.

## Introduction

Superior lead extraction has often been used as the primary approach for pacemaker lead extraction 1. However, approaching lead removal via the femoral vein is necessary in cases where leads cannot be removed via superior lead extraction. Several lead extraction techniques via femoral access have been reported 2. Here, we describe a modified method of transfemoral lead extraction, named as “tetra‐axial” system.

## Case Presentation

**Figure 1:**
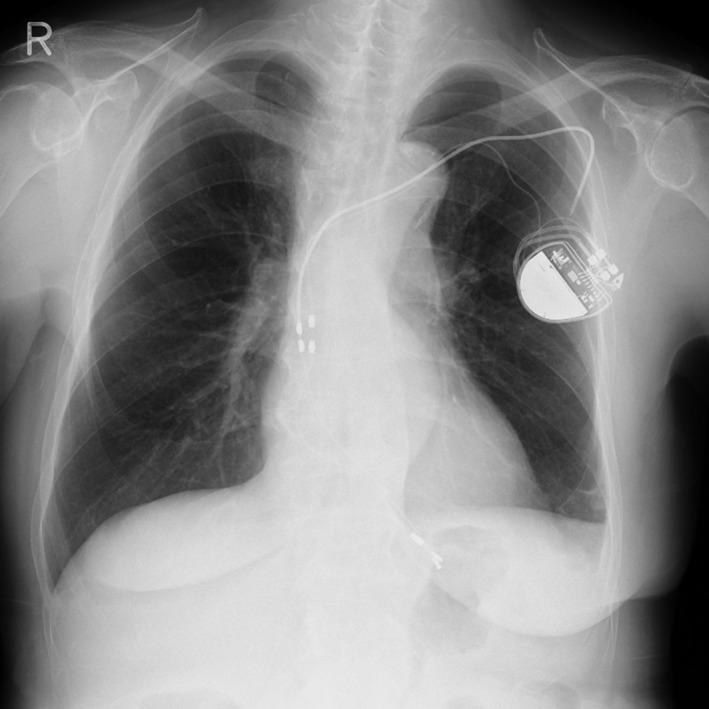
A chest X‐ray revealing the older lead at the patient's left pectoral region, confirming the proximal segment had been cut in a previous procedure, and the newer lead also on her left side.

An 80‐year‐old woman with transient complete atrioventricular block was referred to our institute for remittent fever and mild swelling at her pacemaker pocket site in the left pectoral region, which had been present for 6 months. She was fitted with a single‐lead VDD pacemaker with a passive lead (model 425, Sulzer Intermedics, Angleton, TX) 21 years ago in her left pectoral region. Twelve years before this admission, lead malfunction had occurred, and she had undergone additional lead implantation (model 4345, Sulzer Intermedics), during which the proximal lead segment of the old one was pulled out and cut. Five years before this admission, she had undergone first generator replacement for battery depletion. Physical examination revealed the following vital signs: body temperature, 36.9°C; pulse, 70 beats/min, regular; and blood pressure, 155/70 mm Hg. An electrocardiogram revealed sinus rhythm of 70 beats/min. A chest X‐ray revealed the location of the older lead to be at her left pectoral region, confirming that the proximal segment had been cut in a previous procedure; the newer lead on her left side could also be seen (Fig. 1). Three sets of blood cultures were positive for coagulase‐negative staphylococci. She was diagnosed with lead‐associated endocarditis.

**Figure 2:**
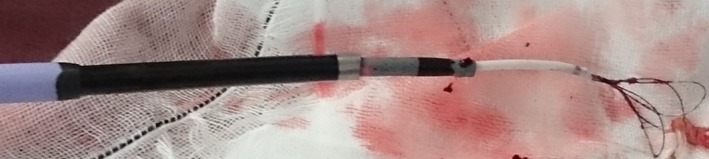
A “tetra‐axial” system consists of a 6‐Fr snare‐loop catheter, an 8.5‐Fr steerable introducer, a 16‐Fr laser sheath, and an 18‐Fr long sheath.

**Figure 3:**
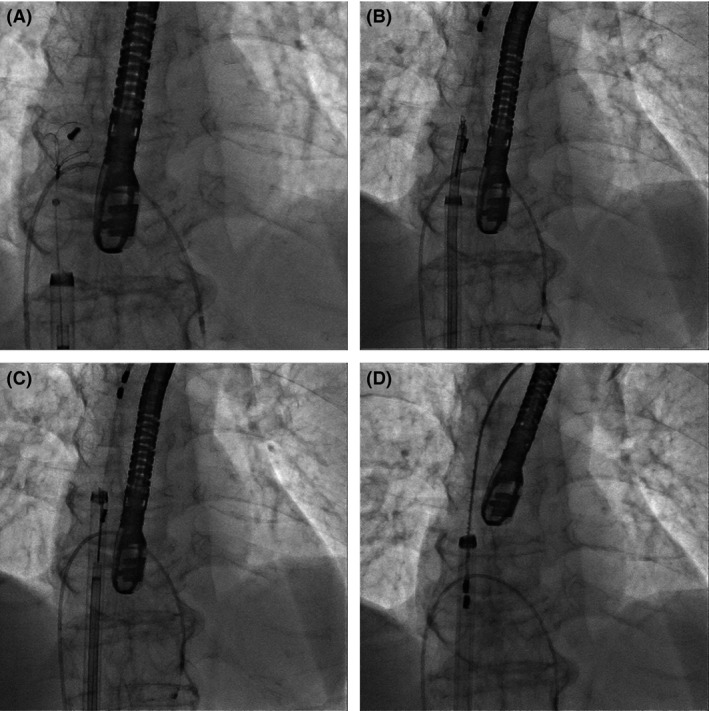
(A) A snare‐loop catheter, with the help of a steerable introducer, catching a pacemaker lead. (B) A 16‐Fr laser sheath coaxially advancing over a steerable introducer. (C) A 18‐Fr sheath coaxially advancing over a 16‐Fr laser sheath. (D) Successful lead extraction into the 18‐Fr sheath.

After a heart team discussion, the two leads were scheduled to be fully extracted by a laser sheath (SLS II, Spectranetics, Colorado Springs, CO). The lead extraction procedure was performed in a hybrid operating room under general anesthesia by both interventional cardiologists and cardiac surgeons, with extracorporeal circulation on standby. Initially, the free end of the newer lead was able to be secured and removed using a locking stylet (Lead Locking Device, Spectranetics) and a 12‐Fr laser sheath. However, the older lead was still adherent to the superior vena cava (SVC), making full removal impossible despite its successful removal from the right ventricle. Instead, mechanical dilation was attempted, but the adhesion of the lead to the SVC was too strong to cut. We then switched to a femoral approach. We first used a 6‐Fr snare‐loop catheter alone to catch the free end of the lead. However, the adhesion was too tight. Therefore, we utilized a “tetra‐axial” system, comprising a 6‐Fr snare‐loop catheter (EN snare, Angiotech Medical Device Technologies, Gainesville, FL), an 8.5‐Fr Agilis NxT steerable introducer (St. Jude Medical, St. Paul, MN), a 16‐Fr laser sheath, and an 18‐Fr long sheath (Fig. 2). After capturing the lead tightly with a snare‐loop catheter, an 8.5‐Fr Agilis NxT steerable introducer was first advanced over a snare‐loop catheter, then a 16‐Fr laser sheath was advanced over, resulting in successful extraction into the 18‐Fr sheath (Fig. 3, Movies [Link], [Link], [Link]). The procedure was completed without any complications. The rest of the patient's stay in the hospital was uneventful, and she was discharged 4 weeks after the procedure.

## Discussion

We described a modified mother–child technique, namely a “tetra‐axial” system, comprising a 6‐Fr snare‐loop catheter, an 8.5‐Fr Agilis NxT steerable introducer, a 16‐Fr laser sheath, and an 18‐Fr long sheath.

It is our opinion that the method we described would be more widely applicable in cases with difficulties in extraction because it has the following advantages: First, our technique enables retrieval of leads in more of a coaxial position and provides a stronger pulling power. Second, aligning the direction of the snare‐loop catheter to the targeted lead is easy with this technique. Sometimes catching a floating lead in the right atrium is difficult, particularly in patients with large ones. However, the Agilis NxT steerable introducer can intentionally direct the snare‐loop catheter to capture a lead, thus shortening the procedure time. Third, the use of a laser sheath would increase the chances of complete extraction. The femoral approach using mechanical dilation has been reported 3. However, laser extraction has the advantage over mechanical dilator extraction in that the former has higher rates of complete removal than the latter 4. Although no reports regarding the efficacy of laser extraction via femoral access have been published, we presume higher rates of complete extraction. Lastly, the caliber of an 18‐Fr sheath can accommodate a 16‐Fr laser sheath. Sometimes, the fibrous tissue encapsulating the extracted doubled‐up leads becomes very large. Therefore, an 18‐Fr size sheath at minimum, in our opinion, is necessary despite the increase of the access‐site bleeding risk.

There are a few considerations to accommodate when employing the “tetra‐axial” system. The risk of thrombus formation within the sheath is one of them. Frequently flushing the sheath with heparinized saline is important. In addition, this technique has the disadvantage of higher costs incurred due to a greater investment in acquiring a 16‐Fr laser sheath. Therefore, this system should be used only when the superior approach is not successful. Finally, the application of a laser sheath in this manner is an absolutely off‐label use. Hence, the “tetra‐axial” system described here should be considered as a last resort.

## Conclusions

A “tetra‐axial” system, consisting of a 6‐Fr snare‐loop catheter, an 8.5‐Fr Agilis NxT steerable introducer, a 16‐Fr laser sheath, and an 18‐Fr long sheath, could become a powerful tool for transfemoral lead extraction, although we have to recognize that the use of a laser sheath in this manner is associated with a potential risk of vascular complications.

## Conflict of Interest

Dr. Yamada has reported speaker fees from DVx Inc. Tokyo, Japan. All other authors have no conflicts of interest to declare.

## Authorship

TI, TH, KY, TO and TY: collected and analyzed the data. TI: wrote the article. TY: had the idea for the article and critically revised it.

## Supporting information


**Movie S1.** A snare‐loop catheter, with the help of a steerable introducer, catching a pacemaker lead.

**Movie S2.** A 16‐Fr laser sheath coaxially advancing over a steerable introducer and a 18‐Fr sheath coaxially advancing over a 16‐Fr laser sheath.

**Movie S3.** Successful lead extraction into the 18‐Fr sheath.
